# Iron Supplementation and Omega-3 Fatty Acids in Tuberculosis: Friend or Foe?

**DOI:** 10.3390/biology15141161

**Published:** 2026-07-16

**Authors:** Marcin Wróblewski, Joanna Wróblewska, Anna Długosz, Celestyna Mila-Kierzenkowska

**Affiliations:** 1 Department of Medical Biology and Biochemistry, Faculty of Medicine, Ludwik Rydygier Collegium Medicum in Bydgoszcz, Nicolaus Copernicus University in Toruń, 24 Karłowicza St., 85-092 Bydgoszcz, Poland; joanna.wroblewska@cm.umk.pl (J.W.); celestyna_mila@cm.umk.pl (C.M.-K.); 2 Department of Bromatology and Pharmacology, Faculty of Chemical Technology and Engineering, Bydgoszcz University of Science and Technology, 3 Seminaryjna St., 85-326 Bydgoszcz, Poland; anna.dlugosz@pbs.edu.pl

**Keywords:** iron, *Mycobacterium tuberculosis*, omega-3 fatty acids, supplementation

## Abstract

Iron supplementation in patients with tuberculosis remains a controversial issue due to the complex role of iron in the host immune response and in the pathogenesis of *Mycobacterium tuberculosis* infection. Although supplementation may be beneficial for some patients with persistent iron deficiency, the available evidence does not support its routine use. The efficacy and safety of such an intervention appear to depend, among other factors, on the activity of the inflammatory process, the severity of iron metabolism disturbances, and the timing of supplementation initiation. Current evidence highlights the need for an individualized assessment of iron status and for further research to establish clear clinical recommendations. At the same time, growing interest has been directed toward the role of long-chain omega-3 polyunsaturated fatty acids, particularly eicosapentaenoic acid (EPA) and docosahexaenoic acid (DHA), as potential modulators of immune and inflammatory responses in tuberculosis. EPA and DHA may influence immune cell function, cytokine production, and the formation of lipid mediators involved in the resolution of inflammation. Experimental evidence suggests that omega-3 fatty acids may reduce the severity of inflammation and infection-associated anemia; however, their effectiveness may depend on baseline fatty acid status, the stage of disease, and interactions with other nutrients, including iron. Human clinical evidence remains very limited and is currently based mainly on one small intervention study, therefore, clinical claims regarding omega-3 fatty acids supplementation should be interpreted cautiously and should not be understood as a recommendation for routine use. The aim of this review is to discuss the significance of iron metabolism and omega-3 fatty acids in tuberculosis, with particular emphasis on their roles in the regulation of inflammation, immune responses, anemia, and potential nutritional support. Current evidence underscores the need for individualized nutritional interventions and further clinical research to determine the safety, efficacy, and optimal timing of iron and omega-3 fatty acids supplementation in patients with tuberculosis.

## Simple Summary

Anemia and disorders of iron metabolism are common among patients with tuberculosis. Available evidence indicates that the efficacy and safety of iron supplementation depend on an individual’s iron status, the severity of inflammation, and the stage of anti-tuberculosis treatment. At the same time, increasing attention is being paid to omega-3 fatty acids, particularly EPA and DHA, which may play a role in regulating inflammation, lipid mediator metabolism, and the resolution of inflammatory processes. Current evidence suggests that both iron supplementation and the potential use of omega-3 fatty acids require careful, individualized assessment, taking into account nutritional status, the severity of inflammation, and the stage of anti-tuberculosis treatment.

## 1. Introduction

Anemia in tuberculosis has a complex etiology. The most common form is anemia of chronic disease, which is associated with persistent inflammation and disturbances in iron metabolism. Other causes of anemia include metabolic disorders, primarily deficiencies of iron, vitamin B12, and folic acid, as well as hemolytic anemia and bone marrow dysfunction [1,2]. Iron deficiency anemia develops primarily as a result of insufficient iron intake and impaired iron absorption. In patients with tuberculosis, these factors may coexist with food insecurity and disease-related loss of appetite [3]. Furthermore, iron deficiency frequently coexists with mechanisms characteristic of anemia of chronic disease, leading to the development of anemia of mixed etiology [3,4]. The coexistence of multiple pathogenic mechanisms may complicate the diagnosis and assessment of the underlying causes of anemia in these patients.

Anemia is a common hematological disorder observed in patients with tuberculosis, with an estimated prevalence ranging from 44% to 89.1% [5]. Among patients with pulmonary tuberculosis, anemia is reported in 20–94% of cases [6]. At the same time, individuals with anemia appear to be at greater risk of developing tuberculosis than those with normal hemoglobin levels. The risk of tuberculosis in patients with mild anemia is at least twofold higher than in individuals without anemia [5]. In addition, anemia is associated with greater disease severity and longer symptom duration, and is particularly common in severe clinical forms such as tuberculous meningitis and disseminated tuberculosis [7,8]. Both iron deficiency anemia and anemia without evidence of iron deficiency have been shown to be associated with more than a twofold increase in the risk of death among patients with tuberculosis [9]. Moreover, the presence of anemia at the initiation of anti-tuberculosis treatment is an independent risk factor for persistent positive sputum smear results after two months of therapy. This risk increases with the severity of anemia, indicating a slower response to anti-tuberculosis treatment [10].

In addition to its role in the development of anemia, iron also plays an important role in the pathogenesis of tuberculosis. This element is involved in both host immune response mechanisms and the metabolic processes of *Mycobacterium tuberculosis*, highlighting the complex relationship between iron homeostasis and the course of infection [11]. In addition to disturbances in iron metabolism, increasing attention has been paid to other nutritional factors that may influence the inflammatory and immune response in tuberculosis. Malnutrition is closely associated with poorer tuberculosis outcomes and may impair host immune competence, emphasizing the importance of nutritional status in disease progression and recovery [12,13]. Emerging clinical evidence suggests that omega-3 fatty acids supplementation may also improve nutritional status during anti-tuberculosis therapy, as reflected by increases in body mass index (BMI) observed in recent intervention studies [14]. Among the nutrients involved in immunometabolic regulation, omega-3 long-chain polyunsaturated fatty acids, particularly eicosapentaenoic acid (EPA) and docosahexaenoic acid (DHA), are of interest for their roles in modulating inflammatory processes, immune cell function, and lipid mediator metabolism [15,16]. Experimental studies in tuberculosis models suggest that EPA and DHA may reduce inflammation, improve anemia of infection, and support host-directed therapeutic strategies; however, their effects may depend on disease stage, baseline omega-3 fatty acids status, and interactions with other nutrients, including iron [11,17]. Therefore, this review aims to discuss the roles of iron metabolism and omega-3 fatty acids in tuberculosis, with particular emphasis on inflammation, immune response, anemia, and nutritional support, as well as the available evidence regarding the potential interactions between these pathways.

## 2. Methodology

This article is a narrative, non-systematic review. The literature was identified through targeted searches of PubMed, Scopus, Web of Science, and Google Scholar, complemented by backward citation searching of relevant reviews and primary studies. Searches focused on combinations of the following terms: tuberculosis, *Mycobacterium tuberculosis*, iron metabolism, hepcidin, ferritin, anemia of inflammation, iron supplementation, omega-3 fatty acids, EPA, DHA, specialized pro-resolving mediators, cytokines, oxidative stress, ferroptosis, and nutritional support. Experimental, observational, and clinical studies were prioritized when they directly addressed tuberculosis, iron homeostasis, omega-3 fatty acids, inflammatory pathways, anemia, or supplementation. Relevant guidelines and policy documents were also considered.

Because the review was not designed as a systematic review, no formal protocol was registered, no exhaustive database-specific search strategy was applied, and study selection was not performed in accordance with PRISMA procedures. The evidence was synthesized narratively with emphasis on mechanistic plausibility, consistency across experimental and clinical data, clinical relevance, and safety considerations. This non-systematic approach allows broad integration of mechanistic and clinical evidence, but it also represents a limitation because selection bias and incomplete retrieval of all available studies cannot be excluded.

## 3. Iron Metabolism and Omega-3 Fatty Acids in the Pathogenesis of Tuberculosis

### 3.1. Iron Metabolism in Tuberculosis

Both inflammation and iron deficiency are considered possible mechanisms underlying anemia in patients with pulmonary tuberculosis. Anemia has been reported to occur in 20–94% of patients with pulmonary tuberculosis [6]. In addition to established risk factors for tuberculosis, such as immunosuppression, HIV infection, diabetes mellitus, malnutrition, and chronic kidney disease, increasing attention has been directed toward the potential role of iron deficiency in the development of this disease. Iron deficiency is the most common nutritional deficiency worldwide and is the leading cause of anemia, which affects approximately one-third of the global population [18]. During tuberculosis, the development of iron deficiency may also be contributed to by secondary factors, such as malnutrition associated with reduced food intake and loss of appetite, malabsorption resulting from inflammation or coexisting infections, and blood loss from hemoptysis [4]. Host immune mechanisms also play an important role in tuberculosis infection. Macrophages are among the first host cells encountered by *M. tuberculosis* and constitute a major intracellular niche for bacterial survival within the host [19].

Iron deficiency is thought to increase susceptibility to tuberculosis by adversely affecting cell-mediated immunity and macrophage function. This relationship is supported by epidemiological studies demonstrating an association between iron deficiency and an increased risk of subsequent tuberculosis [18]. Iron is essential for the proper functioning of the immune system, participating, among other processes, in NK cell activation, T and B lymphocyte proliferation, and the polarization of macrophages toward the M1 phenotype, which is responsible for the pro-inflammatory response [20]. *M. tuberculosis* requires iron for the proper course of metabolic processes essential for its survival and replication within the host organism. In response to infection, the body activates mechanisms that limit iron availability to microorganisms, leading to hypoferremia through iron sequestration in tissues and reduced absorption from the gastrointestinal tract [21]. Iron-sequestering proteins, including lactoferrin, lipocalin-2, transferrin, haptoglobin, and hemopexin, are involved in this process. In tuberculosis, these proteins accumulate, among other sites, within granulomas, where they contribute to limiting iron availability to *M. tuberculosis* and impair mycobacterial growth [4]. During the acute phase of the inflammatory response, increased expression of iron-binding proteins and transporters that remove iron from intracellular compartments occupied by microorganisms also occurs, which constitutes one of the fundamental mechanisms of so-called nutritional immunity [22]. Additionally, lactoferrin released from leukocyte granules during phagocytosis binds iron, preventing its binding to transferrin and thereby impairing iron transport and potentially contributing to the development of anemia associated with tuberculosis [1]. At the site of infection, recruited phagocytes compete with mycobacteria for available iron, further limiting its availability to the microorganisms [4]. Under inflammatory conditions, and in the presence of hepcidin, they increase intracellular iron retention, limiting its availability to extracellular microorganisms; however, they may also create a favorable environment for the proliferation of intracellular mycobacteria [22]. Mechanisms of the inflammatory response affecting iron availability may simultaneously disrupt host iron metabolism. Activation of reticuloendothelial system cells promotes the sequestration of iron outside the pool available for erythropoiesis, which may contribute to the development of anemia associated with chronic inflammation [1].

To survive, *M. tuberculosis* must adapt to the conditions of limited iron availability prevailing within the host organism. The bacilli adapt to the iron-poor environment within the human body by employing a range of specialized mechanisms, including the production of siderophores such as mycobactin and carboxymycobactin. These compounds are characterized by a high capacity to chelate ferric iron, which enables the acquisition of iron from host iron-binding proteins, such as transferrin and ferritin. [21,23]. The synthesis of siderophores is essential for the proliferation of *M. tuberculosis* in both macrophages and the host organism, and disruption of their transport or recycling inhibits mycobacterial growth and constitutes a potential target for novel anti-tuberculosis therapies [22].

Independently of the siderophore system, *M. tuberculosis* can also utilize heme as an alternative source of iron. These bacteria are capable of both endogenous heme synthesis and heme acquisition from the host, with heme serving not only as a source of iron but also as an essential cofactor for numerous cellular processes. It has also been demonstrated that endogenously synthesized heme is more bioavailable to bacteria than heme acquired from the external environment, and that proper heme synthesis plays an important role in the survival of *M. tuberculosis* during macrophage infection [24]. When iron availability becomes limited, *M. tuberculosis* undergoes profound metabolic reprogramming, leading to a transition to a metabolically inactive state (non-replicative persistence) [22].

The importance of iron in the pathogenesis of tuberculosis, however, extends beyond ensuring proper mycobacterial metabolism and the functioning of the host immune system. Iron homeostasis is closely linked to oxidative stress, an important component of the host response to *M. tuberculosis*. Activated macrophages generate reactive oxygen species (ROS) and reactive nitrogen species as part of the antimicrobial response against intracellular bacilli. Although these molecules contribute to bacterial control, excessive oxidative stress may also promote host tissue injury. Because redox-active iron can catalyze ROS generation through the Fenton reaction, disturbances in iron homeostasis may enhance oxidative stress and lipid peroxidation [25]. In this context, studies suggest that *M. tuberculosis* infection may trigger ferroptosis by reducing GPX4 (a selenoprotein, glutathione peroxidase 4) expression and increasing lipid peroxidation in host cells [26]. Ferroptosis has been associated with host cell necrosis and lung tissue damage during TB and may contribute to mycobacterial dissemination. Therefore, oxidative stress and ferroptosis are increasingly recognized as important contributors to *M. tuberculosis* pathogenesis and may represent potential targets for host-directed therapies [25].

### 3.2. Omega-3 Fatty Acids in Tuberculosis

In addition to iron metabolism, other nutritional factors, including omega-3 polyunsaturated fatty acids, may also influence the course of *M. tuberculosis* infection. Bonilla et al. [27] investigated the effects of DHA on the murine macrophage cell line J774A.1 infected with the virulent *M. tuberculosis* H37Rv strain. Compared with the control group, DHA-exposed cells exhibited a higher proportion of infected cells and a greater number of viable bacilli recovered from culture. These findings indicated a reduced ability of the cells to control intracellular *M. tuberculosis* infection. The authors also observed impaired phagolysosome maturation. DHA reduced the presence of markers associated with the late stages of phagolysosome maturation, including lysosome-associated membrane protein 1 (LAMP-1), lysosome-associated membrane protein 3 (LAMP-3), and small GTPase (Rab7), in phagosomes containing *M. tuberculosis*. In addition, DHA-exposed cells showed decreased production of reactive oxygen species (ROS). The authors suggested that impaired phagolysosome maturation and a weakened oxidative response could contribute to the reduced ability of the cells to limit bacterial growth. Similar observations were reported by Jordao et al. [28], who demonstrated that EPA increased the survival of *M. tuberculosis* in infected macrophages, whereas arachidonic acid (AA, omega-6) reduced the number of viable bacteria recovered from the cells. These findings indicated that different fatty acids may influence macrophage responses to *M. tuberculosis* in distinct ways. At the same time, the authors emphasized that relationships observed in cell culture studies do not always translate directly to whole-organism models. In a murine model, an omega-3-enriched diet was associated with a modest but statistically significant reduction in bacterial burden in the lungs and spleen compared with the control group. Further evidence for the complexity of omega-3 fatty acid activity comes from studies conducted in transgenic *fat-1* mice, which are capable of endogenous omega-3 fatty acid synthesis. Following *M. tuberculosis* infection, these animals exhibited higher bacterial loads in the lungs and spleen than control mice. In addition, the lungs of *fat-1* mice showed a less pronounced inflammatory response and more poorly organized granulomatous lesions. The authors suggested that the increased susceptibility of these animals to infection may be partly attributable to an impaired anti-mycobacterial response observed in cells derived from *fat-1* mice [29]. Taken together, these studies indicate that the effects of omega-3 fatty acids on the course of *M. tuberculosis* infection are complex and may depend on the experimental model used. Whereas studies performed in macrophages demonstrated a reduced capacity of these cells to control infection, findings obtained in animal models were not entirely consistent. This suggests that the overall effects of omega-3 fatty acids may depend on interactions among multiple immune mechanisms and on the balance of pro- and anti-inflammatory mediators within the host organism. However, whether these experimental observations can be translated into clinically meaningful benefits in patients with tuberculosis remains uncertain.

Recent clinical evidence provides additional insight into the potential role of omega-3 fatty acids as adjunctive therapy in tuberculosis, although the available human evidence remains very limited and is currently based mainly on a single small intervention study. Ferryansyah et al. [14] conducted a two-group experimental study in 32 patients with drug-sensitive pulmonary tuberculosis receiving standard anti-tuberculosis treatment; 16 patients received adjunctive omega-3 fatty acids supplementation, and 16 served as controls. Omega-3 fatty acids were administered at a dose of 1200 mg/day for 8 weeks, and sputum conversion, BMI, IL-6, and monocyte-to-lymphocyte ratio were assessed at baseline, week 4, and week 8. Adjunctive omega-3 fatty acids supplementation was associated with statistically significant changes in inflammatory and nutritional markers, including IL-6, MLR, and BMI, but did not significantly affect sputum conversion [14]. These findings suggest that the potential benefits of omega-3 fatty acids may primarily result from modulation of inflammatory and immune responses and improvement of overall patient condition rather than from a direct effect on mycobacterial clearance. However, because the evidence is derived from a small study using mainly surrogate endpoints, these results should be interpreted as preliminary and cannot support routine omega-3 fatty acids supplementation in tuberculosis without confirmation in larger, well-designed randomized controlled trials with clinically meaningful endpoints [14].

### 3.3. Interactions Between Iron Metabolism and Omega-3 Fatty Acids in Tuberculosis

Iron metabolism and omega-3 fatty acid metabolism are interconnected, although the mechanisms underlying these interactions remain incompletely understood. Iron is an essential cofactor for desaturase enzymes involved in the biosynthesis of long-chain omega-3 fatty acids, and disturbances in iron homeostasis may impair the conversion of α-linolenic acid into EPA and DHA. Altered iron metabolism has also been associated with changes in tissue EPA and DHA levels and membrane fatty acid composition [30]. Conversely, omega-3 fatty acids may influence iron metabolism by attenuating inflammation and reducing hepcidin expression, thereby indirectly affecting systemic iron homeostasis [11]. Another important point of interaction between iron metabolism and omega-3 fatty acids is lipid peroxidation. Iron overload promotes the generation of ROS, leading to oxidation of polyunsaturated fatty acids. Due to their high degree of unsaturation, EPA and DHA are particularly susceptible to oxidative damage. Consequently, excessive iron-induced lipid peroxidation may contribute to oxidative tissue injury and represents a key feature of ferroptosis [30,31].

## 4. Clinical Significance of Iron Metabolism Markers in Tuberculosis

The role of iron in the development of tuberculosis has been confirmed in both experimental and observational studies. Studies conducted in animal models indicate that iron overload may worsen the course of tuberculosis. Mice with excessive accumulation of this element exhibited a higher bacterial burden in organs, indicating enhanced proliferation of *M. tuberculosis*. Increased iron availability may promote the development of infection and impair host antimicrobial effector mechanisms, such as nitric oxide production [32]. A similar association was also observed in observational studies in humans, in which high dietary iron intake was associated with an increased incidence of active pulmonary tuberculosis [33]. However, it should be emphasized that both iron excess and iron deficiency may adversely affect the course of tuberculosis. Isanaka et al. [9] demonstrated that, among patients with tuberculosis, the presence of iron deficiency without anemia, anemia without iron deficiency, and anemia coexisting with iron deficiency were independently associated with a significantly higher risk of death compared with patients without anemia and iron deficiency.

The importance of iron metabolism in the course of tuberculosis is further supported by studies evaluating ferritin concentration, a widely used marker of iron status. Low ferritin concentration at the initiation of treatment was shown to be independently associated with an increased risk of treatment failure after the first month of therapy. Moreover, among HIV-infected patients, low ferritin concentration was associated with an increased risk of tuberculosis recurrence. In contrast, high ferritin concentration was independently associated with an increased risk of death in the entire study population, including both HIV-infected and HIV-uninfected individuals [34]. These findings complement the observations of Mishra et al. [35], who demonstrated that patients with pulmonary tuberculosis had significantly higher concentrations of ferritin and C-reactive protein (CRP) compared with healthy individuals. Furthermore, a positive correlation was found between ferritin concentration and both disease severity and sputum smear positivity grade, suggesting that this parameter may reflect the intensity of the inflammatory process and disease severity [35]. Ferritin also appears to be the most useful parameter for differentiating anemia of chronic disease from iron deficiency anemia in patients with tuberculosis, outperforming serum iron concentration, total iron-binding capacity (TIBC), and transferrin saturation (TSAT). Elevated ferritin concentration is indicative of anemia of chronic disease, whereas low values are characteristic of iron-deficiency anemia [3]. However, interpreting ferritin concentrations in patients with tuberculosis requires caution. Ferritin is an acute-phase protein; therefore, its elevated concentration may reflect not only increased iron stores but also the intensity of the ongoing inflammatory process. Consequently, assessment of iron status based solely on ferritin concentration may be insufficient and should be supplemented with other indicators of iron metabolism [8]. Interpretation of ferritin concentrations should therefore consider the patient’s inflammatory status and be complemented by other laboratory markers of iron metabolism, particularly TSAT. This approach may help distinguish absolute iron deficiency from anemia of inflammation, in which ferritin concentrations may remain normal or elevated despite restricted iron availability. Measurement of inflammatory markers, such as CRP, may further support the interpretation of ferritin concentrations in the presence of ongoing inflammation [36,37]. In patients with inflammatory conditions, ferritin concentrations > 100 ng/mL together with TSAT < 20% are generally considered indicative of anemia of inflammation [37]. Simultaneous assessment of several iron metabolism parameters, such as ferritin concentration, serum iron, transferrin, and TIBC, allows for a more accurate characterization of disturbances in iron metabolism in patients with active tuberculosis than the analysis of a single marker [8,38]. Among the additional parameters used to assess iron metabolism, soluble transferrin receptor (sTfR) concentration may be useful in identifying iron deficiency in patients with pulmonary tuberculosis and concomitant inflammation [39]. However, it should be noted that sTfR measurement is not currently recommended for the routine diagnosis of iron deficiency [40]. Increasing interest has also been directed toward regulators of iron homeostasis, such as hepcidin and ferroportin, which may serve as potential biomarkers of infection activity and progression; however, their application in clinical practice requires further validation [41].

Hepcidin, the principal hormone regulating iron metabolism, plays an important role in the development of anemia of inflammation. In tuberculosis, elevated hepcidin concentrations enhance iron sequestration in macrophages and inhibit both intestinal iron absorption and iron release from body stores, thereby reducing iron availability for erythropoiesis [21]. It has been suggested that low hepcidin concentration may indicate true iron deficiency, whereas elevated hepcidin levels are characteristic of anemia of chronic disease associated with tuberculosis and persistent inflammation [2]. Despite the growing interest in hepcidin as a biomarker of iron metabolism disorders, its routine clinical use remains limited. The main challenges include the lack of assay harmonization and universally accepted reference ranges, which hinder comparison of results between laboratories and complicate their clinical interpretation. Various analytical techniques, primarily immunoassays and mass spectrometry-based methods, have been used to quantify hepcidin, but they often yield substantially different absolute concentrations, largely due to differences in assay calibration, highlighting the need for assay harmonization. Furthermore, the lack of clinically approved assays currently restricts the use of hepcidin measurement as a diagnostic biomarker in routine clinical practice [42,43]. This limitation is particularly relevant in the context of tuberculosis, which disproportionately affects low- and middle-income countries where access to specialized laboratory assays is often limited. Therefore, despite its promising diagnostic and prognostic potential, the widespread clinical implementation of hepcidin as a biomarker in tuberculosis will require the development of standardized, accessible, and affordable assays. An overview of studies investigating the role of iron metabolism biomarkers in tuberculosis is presented in Table 1.

The prognostic interpretation of ferritin and hepcidin concentrations also requires caution. Both biomarkers are strongly influenced by the inflammatory response. Serum ferritin is a well-recognized acute-phase reactant and may increase in a wide range of inflammatory conditions, infections, and malignancies independently of body iron stores. Likewise, inflammation, particularly that driven by interleukin-6 (IL-6), induces hepatic hepcidin synthesis, resulting in iron sequestration and reduced iron availability for erythropoiesis. Therefore, elevated ferritin and hepcidin concentrations observed in patients with tuberculosis may reflect both alterations in iron metabolism and the intensity of the underlying inflammatory response. Consequently, associations between these biomarkers and the clinical course of tuberculosis should be interpreted with caution, as the contribution of inflammation may complicate the assessment of their independent prognostic value [47,48].

## 5. Cytokines, Iron Homeostasis, and Omega-3-Derived Lipid Mediators in Tuberculosis

### 5.1. Cytokine-Mediated Regulation of Iron Homeostasis in Tuberculosis

Effective control of mycobacterial infections requires the coordinated action of innate, cellular, and humoral immune mechanisms, regulated by numerous cytokines. Macrophages involved in phagocytosis and CD4+ T lymphocytes, responsible for the induction and maintenance of the granulomatous response, play key roles in host defense. Impairment of these mechanisms, observed for example in individuals with untreated HIV infection, increases susceptibility to mycobacterial infections. The nature of the infection and its clinical course depend on both host-related factors and the mycobacterial species responsible for the disease [49].

In tuberculosis, increased hepcidin synthesis is induced by pro-inflammatory cytokines, primarily IL-6, as well as interleukin-1 (IL-1), interleukin-22 (IL-22), and interferon gamma (IFN-γ). In active tuberculosis, pro-inflammatory signals, particularly those mediated by IL-6, predominate over erythropoietic stimuli, leading to persistently elevated hepcidin levels [4,21]. Inflammatory cytokines may also exacerbate hypoferremia independently of hepcidin induction. IFN-γ promotes the differentiation of myeloid cells at the expense of erythropoiesis and activates macrophages. Tumor necrosis factor alpha (TNF-α) inhibits erythroid cell proliferation and may also reduce intestinal iron absorption through its effects on duodenal cells. IL-1, IL-6, interleukin-10 (IL-10), and TNF-α also influence macrophage iron metabolism by modulating the expression of proteins involved in iron transport, uptake, and storage, thereby promoting iron retention within cells of the phagocytic system [4]. IL-6 concentrations are significantly higher in patients with active tuberculosis than in healthy individuals, with particularly elevated levels observed in patients with anemia and more advanced disease. Following successful chemotherapy, IL-6 levels decrease, whereas hemoglobin and IFN-γ levels increase. Moreover, higher IL-6 concentrations correlate with greater tuberculosis severity and lower hemoglobin and IFN-γ levels, suggesting a role for IL-6 in the pathogenesis of anemia of inflammation and disease progression [50]. Elevated levels of interleukin-1 beta (IL-1β) and interleukin-7 (IL-7) have also been observed in patients with tuberculosis and iron deficiency. It has been proposed that increased concentrations of both cytokines may reflect enhanced inflammation and represent a compensatory mechanism associated with the reduced number of unconventional T lymphocytes observed in iron-deficient patients. IL-1β plays an important role in antimycobacterial protection during the early stage of infection; however, its prolonged production has been associated with neutrophil infiltration, disease progression, and poorer treatment outcomes. In contrast, IL-7 participates in T-cell lymphopoiesis and supports the survival of memory T cells [20]. Beyond these roles, IL-7 supports the survival and maintenance of T-cell populations, including memory T-cell subsets, which are important for long-term immunity against *M. tuberculosis*. In addition, unconventional T lymphocytes bridge innate and adaptive immunity by rapidly producing IFN-γ and other cytokines that enhance macrophage antimicrobial activity and contribute to the control of *M. tuberculosis* infection [51,52].

In contrast to many chronic diseases, in which reducing inflammation is generally considered beneficial, the inflammatory response in tuberculosis plays a dual role. Although excessive inflammation can lead to tissue damage, a properly regulated pro-inflammatory response is essential for limiting *M. tuberculosis* replication and for maintaining granuloma integrity. Consequently, excessive suppression of the immune response may impair the host’s ability to control the infection and may facilitate the dissemination of mycobacteria [19].

### 5.2. Omega-3-Derived Lipid Mediators and Resolution of Inflammation in Tuberculosis

**Figure 1:**
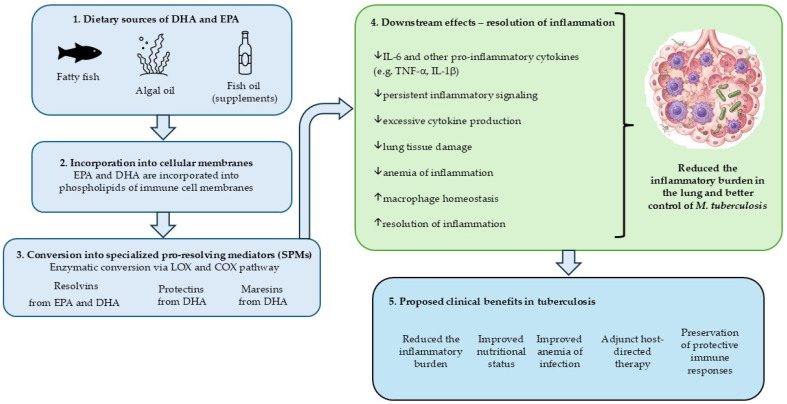
Proposed mechanisms linking dietary EPA and DHA with immune modulation, specialized pro-resolving mediator formation, cytokine regulation, and inflammatory burden in tuberculosis. IL—interleukin; TNF-α—tumor necrosis factor alfa, ↑ increase, ↓ decrease.

Omega-3 long-chain polyunsaturated fatty acids may also participate in the regulation of cytokine-mediated inflammation in tuberculosis. EPA and DHA influence immune cell function, in part through their incorporation into cell membranes and in part through their conversion into lipid mediators involved in the resolution of inflammation. EPA and DHA are precursors of specialized pro-resolving mediators, including resolvins, protectins, and maresins, which actively promote the resolution of inflammation, limit excessive neutrophil recruitment, support macrophage efferocytosis, and may contribute to restoration of tissue homeostasis [16]. In tuberculosis, this mechanism may be relevant because excessive or prolonged production of pro-inflammatory cytokines contributes not only to antimicrobial defense, but also to tissue injury, wasting, and anemia of infection. Therefore, omega-3 fatty acids may affect the balance between protective cellular immunity and inflammation-related pathology [16]. Figure 1 illustrates the proposed mechanisms linking dietary EPA and DHA with immune modulation, specialized pro-resolving mediator (SPM) formation, cytokine regulation, and the inflammatory burden in tuberculosis.

Experimental studies indicate that n-3 long-chain polyunsaturated fatty acids may modulate cytokine-related inflammatory activity during *M. tuberculosis* infection. In infected C3HeB/FeJ mice, EPA/DHA supplementation reduced systemic and pulmonary inflammation and attenuated anemia of infection. These effects were accompanied by changes in cytokine-mediated inflammatory responses; however, the benefits were reduced when EPA/DHA were administered together with iron, suggesting an interaction between fatty acid metabolism, iron status, and inflammatory regulation [11]. This observation is important in the context of tuberculosis, where IL-6-driven hepcidin synthesis, macrophage iron retention, and inflammatory anemia are closely interrelated. Recent clinical evidence also supports the immunomodulatory role of omega-3 fatty acids in tuberculosis. In a small two-group intervention study involving patients with drug-sensitive pulmonary tuberculosis, supplementation with 1200 mg/day of omega-3 fatty acids was associated with a significant reduction in IL-6 concentrations during anti-tuberculosis treatment, suggesting that omega-3 fatty acids may exert immunomodulatory effects in humans; however, these findings should be interpreted as preliminary [14].

The influence of omega-3 fatty acids on inflammation in tuberculosis should be interpreted as immunomodulatory rather than simply anti-inflammatory. Effective control of *M. tuberculosis* requires the activation of macrophages and T-cell-mediated immune responses, including the production of cytokines such as IFN-γ and TNF-α. At the same time, persistent inflammatory activation may intensify lung tissue damage and systemic metabolic disturbances. By supporting the formation of specialized pro-resolving lipid mediators, omega-3 fatty acids may contribute to the active resolution of inflammation without necessarily suppressing host defense mechanisms. This concept is consistent with host-directed therapeutic strategies, in which modulation of lipid mediator metabolism is considered a potential adjunctive approach in tuberculosis [16,17].

Further experimental data suggest that the effect of n-3 long-chain polyunsaturated fatty acids (LCPUFA) treatment may depend on the baseline omega-3 fatty acid status of the host. In a tuberculosis mouse model, adjunctive n-3 LCPUFA treatment was associated with reduced cytokine-mediated inflammation and improved anemia of infection, particularly in animals with low initial n-3 fatty acid status [53]. These findings suggest that omega-3 fatty acids may influence cytokine-driven inflammation and iron-related metabolic disturbances in tuberculosis, although their clinical relevance in humans remains to be confirmed [11,53]. Therefore, future studies should evaluate not only cytokine concentrations, but also hepcidin, ferritin, TSAT, lipid mediator profiles, and clinically meaningful outcomes to determine whether omega-3 fatty acids supplementation can beneficially modulate inflammation-driven iron disturbances in tuberculosis [11,14,16,17,53].

## 6. Safety and Efficacy of Iron and Omega-3 Fatty Acid Supplementation in Tuberculosis

### 6.1. Safety and Efficacy of Iron Supplementation in Tuberculosis

Treatment of tuberculosis alone leads to a significant reduction in the prevalence of anemia of inflammation; however, it does not completely eliminate all forms of anemia. The persistence of iron-deficiency anemia, as well as anemia with mixed inflammatory and deficiency etiologies, may indicate the need for additional interventions to improve iron homeostasis [54]. Nevertheless, according to the latest WHO guidelines, routine supplementation with micronutrients, including iron, is not recommended for patients with tuberculosis in the absence of confirmed deficiencies and clear evidence of clinical benefit from such an intervention [55]. This approach is justified by the complex role of iron in the pathogenesis of tuberculosis. Experimental studies have demonstrated that limited iron availability may inhibit the proliferation of *M. tuberculosis*; however, it does not lead to bacterial eradication. On the contrary, such conditions may promote the transition of bacteria into a persistent state, enabling long-term survival of the pathogen and potentially sustaining chronic or latent tuberculosis infection in some infected individuals. Furthermore, it has been shown that *M. tuberculosis* bacilli remaining in a growth-arrested state due to iron deficiency exhibit reduced susceptibility to isoniazid, a first-line anti-tuberculosis drug also used to treat latent tuberculosis infection [56]. These findings suggest that both iron excess and iron deficiency may influence the course of tuberculosis. Therefore, assessment of iron status and decisions regarding iron supplementation should be made on an individual basis, taking into account the patient’s clinical condition and laboratory findings.

Despite concerns about the potential impact of iron on the course of infection, findings from some studies suggest that iron supplementation may confer clinical benefits in selected patient populations. In patients with spinal tuberculosis and concomitant iron deficiency, iron supplementation administered alongside anti-tuberculosis therapy was associated with favorable changes in immune response parameters, including a significant increase in IFN-γ levels and a reduction in sTfR concentrations. However, these findings are derived from a small study involving only patients with spinal tuberculosis and therefore require confirmation in larger studies [57].

Alterations in iron metabolism observed during tuberculosis suggest that the timing of iron supplementation may influence its effectiveness. Elevated hepcidin levels associated with active inflammation may limit both intestinal iron absorption and the release of iron from macrophages, thereby potentially reducing the efficacy of iron supplementation. Monitoring biomarkers of iron metabolism may be useful in assessing tuberculosis-associated changes in iron homeostasis and potentially in identifying individuals at increased risk of an unfavorable disease course [54]. However, further studies are needed to determine whether these biomarkers can be used to identify the optimal timing for initiating iron supplementation. Cercamondi et al. [21] demonstrated that patients with active tuberculosis had a markedly reduced capacity to absorb iron from the gastrointestinal tract. The authors attributed this phenomenon to chronic inflammation, which, through increased hepcidin concentrations, led to iron sequestration and impaired intestinal iron absorption. During anti-tuberculosis treatment, a gradual reduction in inflammation, a decrease in hepcidin levels, and an improvement in hematological parameters were observed, despite the absence of iron supplementation. These changes were accompanied by improved iron availability for erythropoiesis, promoting the recovery of red blood cell production and a gradual increase in hemoglobin concentration. Following completion of treatment, iron absorption improved substantially, reaching a level that allowed its effective utilization by the body. At the same time, most patients showed marked improvement in hematological parameters and the resolution of anemia without iron supplementation. Based on these findings, the authors concluded that iron supplementation before or during tuberculosis treatment is likely to be ineffective and, in most cases, unnecessary. They further suggested that iron supplementation should primarily be considered in patients whose anemia persists after completion of anti-tuberculosis therapy. Moreover, the authors noted that even after the intensive phase of treatment, iron absorption remained impaired, which does not support the routine initiation of iron supplementation at that stage of therapy. These findings are consistent with the observations of Devi et al. [58], who demonstrated that iron supplementation accelerated increases in hemoglobin concentration, hematocrit, and mean corpuscular volume during the early phase of treatment. However, long-term improvement in hematological parameters was primarily associated with the effectiveness of anti-tuberculosis therapy.

In addition to the limited evidence on the efficacy of iron supplementation, the safety of this intervention remains a concern. Some insight into this issue is provided by a case report published by Karakonstantisa et al. [59]. The authors described a patient with Crohn’s disease receiving adalimumab therapy who developed reactivation of central nervous system tuberculosis within one week of intravenous administration of ferric carboxymaltose. Given the presence of other risk factors for tuberculosis reactivation, including treatment with a TNF-α inhibitor and incomplete treatment of latent tuberculosis infection, the specific role of iron supplementation in the development of the disease cannot be determined with certainty. Nevertheless, the authors emphasized that the close temporal relationship between iron administration and symptom onset, together with the biological plausibility of this association, warrants further investigation into the potential impact of intensive iron supplementation on the reactivation of latent tuberculosis infection in patients with additional risk factors.

Additional insights are provided by the experimental study conducted by Kolloli et al. [60]. The study employed a rabbit model of *Mycobacterium tuberculosis* infection in which, following an initial phase of active infection, bacterial replication became controlled, and the infection persisted without progression to active disease. The authors demonstrated that iron supplementation did not significantly affect either the bacterial burden or the severity of pathological changes in the lungs, regardless of the timing of its administration. However, given the use of an animal model, these findings require confirmation in clinical studies.

At present, there are no studies evaluating the effects of iron supplementation in individuals who have been in close contact with patients with tuberculosis. However, available evidence suggests that disturbances in iron metabolism may precede the development of active tuberculosis. It remains unclear whether modifying iron status through supplementation influences the risk of developing tuberculosis; therefore, routine iron supplementation in this population is not currently justified and requires further investigation.

### 6.2. Safety and Efficacy of Omega-3 Fatty Acid Supplementation in Tuberculosis

In contrast to iron supplementation, omega-3 fatty acid supplementation in tuberculosis is considered mainly in the context of its potential immunomodulatory and inflammation-resolving effects. EPA and DHA are precursors of specialized pro-resolving lipid mediators and may influence the balance between protective immune activation and excessive inflammation-related tissue damage. This mechanism is particularly relevant in tuberculosis, where persistent inflammation contributes to lung pathology, anemia of infection, and systemic metabolic disturbances [16].

Further studies have evaluated n-3 long-chain polyunsaturated fatty acids as adjunctive host-directed therapy during anti-tuberculosis treatment. Longer-term n-3 LCPUFA supplementation in a C3HeB/FeJ tuberculosis mouse model was associated with improved inflammation-resolving and anti-inflammatory responses and appeared more favorable than ibuprofen as an adjunctive approach in this experimental setting [17]. Other findings suggest that the response to n-3 LCPUFA treatment may depend on the host’s baseline omega-3 fatty acid status, with potential effects on cytokine-mediated inflammation and anemia of infection [53].

Recent clinical evidence provides additional insight into the potential role of omega-3 fatty acids as adjunctive therapy in tuberculosis. In patients with drug-sensitive pulmonary tuberculosis, omega-3 fatty acids supplementation was associated with a reduction in the monocyte-to-lymphocyte ratio (MLR), a marker increasingly investigated as an indicator of immune activation and disease activity, as well as favorable changes in inflammatory parameters, including lower IL-6 concentrations, and improvements in nutritional status reflected by increased BMI. Despite these beneficial effects, omega-3 fatty acids supplementation did not significantly affect sputum conversion rates. These findings suggest that the potential benefits of omega-3 fatty acids may primarily result from modulation of inflammatory and immune responses and improvement of overall patient condition rather than from a direct effect on mycobacterial clearance [14].

Most of the currently available evidence originates from preclinical animal studies. In a C3HeB/FeJ mouse model, adjunctive EPA/DHA supplementation during standard anti-tuberculosis therapy promoted a more balanced Th1/Th2 immune response, attenuated excessive Th17-mediated inflammation, and was associated with lower bacterial burden and reduced lung inflammation compared with adjunctive ibuprofen. Similarly, an experimental study conducted in 40 male albino rats demonstrated that omega-3 fatty acids attenuated anti-tuberculosis drug-induced hepatotoxicity, nephrotoxicity, and hematological abnormalities. Although these findings are encouraging, they cannot be directly extrapolated to humans. A Phase II randomized clinical trial (TREAT-3) evaluating EPA/DHA supplementation as adjunctive therapy in adults with drug-susceptible pulmonary tuberculosis is currently ongoing in South Africa. The results of this study are not yet available and are expected to provide important evidence regarding the safety and efficacy of omega-3 supplementation during anti-tuberculosis treatment [61,62,63].

Despite these promising observations, current evidence remains insufficient to recommend routine omega-3 fatty acids supplementation for all patients with tuberculosis. Most available data come from experimental animal models, whereas well-designed clinical trials in humans are lacking. Therefore, omega-3 fatty acids should currently be considered a potential adjunctive nutritional strategy requiring further clinical validation. Future studies should determine the optimal dose, duration, and timing of supplementation, assess its safety during standard anti-tuberculosis therapy, and identify patient groups that may benefit most, particularly those with malnutrition, low dietary fish intake, increased inflammatory burden, or low baseline omega-3 fatty acids status [16,17,53].

### 6.3. Evidence on Omega-3 Fatty Acids–Iron Interactions in Tuberculosis and Related Inflammatory Conditions

Experimental evidence suggests that omega-3 fatty acid supplementation may have beneficial effects on inflammation and infection-related anemia in tuberculosis models. In *M. tuberculosis*-infected mice, post-infection EPA/DHA supplementation reduced systemic and pulmonary inflammation, improved anemia of infection, and was associated with lower lung bacterial burden when administered separately from iron. However, these favorable effects were attenuated when EPA/DHA were combined with iron supplementation, indicating that combined nutritional interventions cannot be assumed to have additive effects and should be carefully evaluated. Importantly, these findings were obtained in a murine model of tuberculosis and have not been confirmed in clinical studies. Therefore, no conclusions can currently be drawn regarding the safety or efficacy of combined iron and omega-3 fatty acids supplementation in patients with tuberculosis, highlighting the need for dedicated clinical trials [11].

As this is the only available study evaluating the combined effects of omega-3 fatty acid and iron supplementation in a tuberculosis model, indirect evidence from studies conducted in other patient populations may provide additional context for interpreting these findings. However, the results of these studies cannot be directly extrapolated to tuberculosis. Fujibayashi et al. [64] demonstrated that EPA and DHA supplementation in young women with iron deficiency increased serum ferritin concentrations without affecting hemoglobin, hepcidin, or inflammatory markers, suggesting a potential improvement in iron stores, although the underlying mechanism remains unclear and requires further investigation. In contrast, Uyoga et al. [65] observed that three months of EPA/DHA supplementation did not improve iron absorption or reduce hepcidin concentrations and inflammatory markers in women with overweight or obesity, despite a significant increase in the omega-3 index, which nevertheless remained below the optimal range. Similarly, Gharekhani et al. [66] reported that four months of omega-3 fatty acids supplementation in maintenance hemodialysis patients did not significantly improve anemia-related outcomes or most markers of iron metabolism, although it was associated with modest anti-inflammatory effects, including a significantly smaller increase in serum ferritin concentrations and an improved IL-10/IL-6 ratio compared with placebo. Collectively, these findings indicate that the effects of omega-3 fatty acids on iron metabolism are context-dependent and appear to vary according to the underlying disease, and inflammatory status.

The available clinical studies do not provide a direct explanation for the attenuation of the beneficial effects observed when EPA/DHA and iron were administered together in the murine tuberculosis model. Nevertheless, evidence from an experimental model of diabetes suggests that iron may enhance oxidative stress and lipid peroxidation, potentially diminishing the anti-inflammatory effects of omega-3 fatty acids [67]. Whether similar mechanisms operate during *M. tuberculosis* infection remains unknown and requires further mechanistic investigation.

## 7. Clinical and Research Implications

The available evidence does not support routine iron supplementation in all patients with tuberculosis [55]. In clinical practice, iron supplementation should be considered only after individualized assessment of iron status, inflammatory activity, anemia type, and stage of anti-tuberculosis treatment [21,54,55]. Ferritin should not be interpreted as a single marker of iron status because it may increase as an acute-phase reactant during active inflammation. Therefore, assessment of iron metabolism should preferably include a combination of biomarkers, such as ferritin, TSAT, serum iron, transferrin or TIBC, and inflammatory markers such as CRP [3,8,38,40]. Omega-3 fatty acids should currently be regarded as a potential adjunctive nutritional strategy rather than a routinely recommended intervention in tuberculosis [14,16,17,53]. Their possible benefits may be related mainly to modulation of inflammation, lipid mediator metabolism, and improvement of nutritional status, but the available human evidence remains very limited and is currently based mainly on one small intervention study [14]. Therefore, omega-3 fatty acids supplementation should not be routinely recommended until larger, well-designed clinical trials confirm its safety and efficacy [14,16,17,53]. Combined iron and omega-3 fatty acids supplementation requires particular caution because experimental evidence suggests that the beneficial effects of EPA/DHA may be attenuated when omega-3 fatty acids are co-administered with iron [11]. Thus, combined supplementation should not be assumed to have additive effects or to be clinically established. Future studies should evaluate clinically meaningful outcomes, including treatment response, sputum conversion, anemia resolution, nutritional recovery, safety, and mortality, as well as biomarkers of iron metabolism, inflammation, oxidative stress, and omega-3-derived lipid mediator profiles [11,14,16,17,53,54].

## 8. Conclusions

Iron metabolism plays a central role in the pathogenesis and clinical course of tuberculosis, influencing both host immune responses and the survival of *Mycobacterium tuberculosis*. Disturbances in iron homeostasis, including iron deficiency, anemia of inflammation, and iron overload, are associated with adverse clinical outcomes, such as increased disease severity, delayed treatment response, and higher mortality risk. Current evidence does not support routine iron supplementation in patients with tuberculosis. The potential benefits of supplementation appear to depend on individual iron status, the degree of inflammation, and the stage of anti-tuberculosis treatment. Therefore, assessment of iron metabolism should be individualized and based on a combination of biomarkers rather than a single parameter.

Omega-3 fatty acids, particularly EPA and DHA, represent a promising adjunctive nutritional strategy due to their ability to modulate inflammatory responses and promote the resolution of inflammation. Experimental evidence suggests that omega-3 fatty acids supplementation may reduce inflammatory burden, improve anemia of infection in selected tuberculosis models, whereas human clinical evidence remains limited mainly to one small intervention study reporting changes in IL-6, MLR and BMI. However, the effects of omega-3 fatty acids appear to depend on baseline nutritional status, disease stage, timing of supplementation and potential interactions with iron metabolism. In particular, the attenuation of EPA/DHA benefits after co-administration with iron in experimental tuberculosis models indicates that combined supplementation should not be assumed to have additive effects or to be clinically established. At present, the available evidence remains insufficient to recommend routine supplementation with either iron or omega-3 fatty acids in all patients with tuberculosis. Future well-designed clinical trials are needed to clarify their safety, efficacy, optimal timing, clinically meaningful endpoints, and the patient populations most likely to benefit from these interventions.

## Figures and Tables

**Table 1:** Iron metabolism biomarkers in tuberculosis: associations with infection, progression, and clinical outcomes.

Patient Characteristics	Key Findings	Progression to Active TB	Conclusions	Ref.
*n* = 150 (TB patients: 50; household contacts: 50; healthy controls: 50)	↑ ferritin and hepcidin levels; ↓ transferrin and hemoglobin levels; trend toward lower ferroportin expression in TB patients	Not prospectively assessed	Active tuberculosis is associated with altered iron homeostasis; hepcidin and ferritin may represent potential biomarkers of disease activation	[41]
*n* = 200 (household contacts: 98; TB patients: 102)	↑ hepcidin, ferritin, CRP, and procalcitonin levels; ↓ transferrin, hemoglobin, and serum iron levels in TB patients	5 individuals developed active TB. These individuals had elevated hepcidin levels before disease onset	Hepcidin may serve as a biomarker of TB severity and may identify TB-exposed individuals at increased risk of developing active disease	[2]
*n* = 72 (27 household contacts; 45 active TB patients)	↑ hepcidin and ferritin levels; ↓ transferrin levels in active TB patients	10 household contacts progressed to active TB; ↓ baseline transferrin was associated with progression, while early progressors showed ↑ hepcidin and ferritin levels	Iron homeostasis biomarkers (transferrin, ferritin and hepcidin) may help identify household contacts at increased risk of progression to active TB	[44]
*n* = 70 (35 TB patients; 35 household contacts)	↓ serum iron and hemoglobin levels in TB patients; positive correlation between serum iron levels and *NRAMP1* gene expression	Not assessed	Active TB is associated with altered iron homeostasis and decreased *NRAMP1* gene expression	[45]
*n* = 430 (household contacts)	↑ ferritin levels were associated with an increased risk of *M. tuberculosis* infection among household contacts.	115 participants developed new *M. tuberculosis* infection	Iron metabolism may play an important role in the early stages of *M. tuberculosis* infection	[46]

*n*—number of participants; *NRAMP1*—gene of natural resistance-associated macrophage protein 1; TB—tuberculosis; ↑—increase; ↓—decrease.

## Data Availability

No new data were created or analyzed in this study. Data sharing is not applicable to this article.

## Abbreviations

The following abbreviations are used in this manuscript:

AA	Arachidonic acid
BMI	Body mass index
CRP	C-reactive protein
DHA	Docosahexaenoic acid
EPA	Eicosapentaenoic acid
GPX4	Glutathione peroxidase 4
IDA	Iron deficiency anemia
IFN-γ	Interferon gamma
IL	Interleukin
LAMP-1	Lysosome-associated membrane protein 1
LAMP-3	Lysosome-associated membrane protein 3
LCPUFA (n-3)	Long-chain polyunsaturated fatty acids (omega-3 fatty acids)
MLR	Monocyte-to-lymphocyte ratio
*NRAMP1*	Gene of natural resistance-associated macrophage protein 1
Rab7	Small GTPase
ROS	Reactive oxygen species
sTfR	Soluble transferrin receptor
TB	Tuberculosis
TIBC	Total iron-binding capacity
TNF-α	Tumor necrosis factor alpha
TSAT	Transferrin saturation
